# Does ultrasound education improve anatomy learning? Effects of the Parallel Ultrasound Hands-on (PUSH) undergraduate medicine course

**DOI:** 10.1186/s12909-022-03255-4

**Published:** 2022-03-27

**Authors:** Wei-Ting Chen, Yi-No Kang, Ting-Cheng Wang, Che-Wei Lin, Chung-Yi Cheng, Fat-Moon Suk, Chin-Wang Hsu, Sha-Ku Huang, Wen-Cheng Huang

**Affiliations:** 1 Department of Emergency, School of Medicine, College of Medicine, Taipei Medical University, No.111, Sec. 3, Xinglong Rd, Taipei City, 11696 Taiwan; 2 Department of Emergency and Critical Medicine, Taipei Municipal Wan-Fang Hospital, Taipei Medical University, Taipei City, Taiwan; 3 Department of Education and Humanities in Medicine, School of Medicine, College of Medicine, Taipei Medical University, Taipei City, Taiwan; 4 Department of Education, Taipei Municipal Wan-Fang Hospital, Taipei Medical University, Taipei City, Taiwan; 5 Evidence-Based Medicine Center at Taipei Medical University, Taipei City, Taiwan; 6 Center for Education in Medical Simulation, Taipei Medical University, Taipei City, Taiwan; 7 Department of Internal Medicine, School of Medicine, College of Medicine, Taipei Medical University, Taipei City, Taiwan; 8 Division of Nephrology, Department of Internal Medicine, Wan Fang Hospital, Taipei Medical University, Taipei City, Taiwan; 9 Division of Gastroenterology, Department of Internal Medicine, Wan Fang Hospital, Taipei Medical University, Taipei City, Taiwan; 10 Research Center for Environmental Medicine, Kaohsiung Medical University, Sanmin, Kaohsiung, Taiwan; 11 National Institute of Environmental Health Sciences, National Health Research Institutes, Zhunan Township, Miaoli County, Taiwan

**Keywords:** Gross anatomy education, Medical education, Undergraduate education ultrasound education, Parallel ultrasound course

## Abstract

**Background:**

As ultrasound has become increasingly prominent in medicine, portable ultrasound is perceived as the visual stethoscope of the twenty-first century. Many studies have shown that exposing preclinical students to ultrasound training can increase their motivation and ultrasound competency. However, few studies have discussed the effect of ultrasound training on anatomy learning.

**Method:**

The Parallel Ultrasound Hands-on (PUSH) course was designed to investigate whether or not ultrasonography training affects anatomy knowledge acquisition. The PUSH course included anatomical structures located in the chest and abdomen (target anatomy) and was conducted in parallel to the compulsory gross anatomy course.

Learners (*n* = 140) voluntarily participated in this elective course (learners in the course before the midterm examination (Group 1, *n* = 69), or after the midterm examination (Group 2, *n* = 71)). Anatomy examination scores (written and laboratory tests) were utilized to compare the effects of the PUSH course.

**Result:**

Group 1 obtained significantly higher written test scores on the midterm examination (mean difference [MD] = 1.5(7.6%), *P* = 0.014, Cohen’s *d* = 0.43). There was no significant difference in the final examination scores between the two groups (Written Test: MD = 0.3(1.6%), *P =* 0.472). In Laboratory test, both mid-term (MD:0.7(2.8%), *P* = 0.308) and final examination (MD:0.3(1.5%), *P =* 0.592) showed no significant difference between two groups. Students provided positive feedback in overall learning self-efficacy after the PUSH course (Mean = 3.68, SD = ±0.56 on a 5-point Likert scale). Learning self-efficacy in the cognitive domain was significantly higher than that in the affective domain (MD = 0.58; *P* < 0.001) and psychomotor domain (MD = 0.12; *P* = 0.011).

**Conclusion:**

The PUSH course featured a hands-on learning design that empowered medical students to improve their anatomy learning.

**Supplementary Information:**

The online version contains supplementary material available at 10.1186/s12909-022-03255-4.

## Background

Ultrasound is an important and indispensable technology in medicine. Due to its nonradiative and noninvasive nature, ultrasound has long been used in specialties such as radiology, obstetrics/gynecology, and cardiology. There are increasingly more ultrasound applications in specialties such as emergency medicine and critical care medicine [1, 2]. Because ultrasound is vital to clinical practice, it should be taught and emphasized in medical education. Portable ultrasound came to be seen as the visual stethoscope [3] and it started to be used as a tool in learning anatomy [4–9]. Many studies have shown that exposing undergraduate students to ultrasound can increase their interest, ultrasound skills and image recognition ability [4, 10]. It is reasonable that students improve their ultrasound knowledge after taking a course with an ultrasound curriculum. Several studies have also discussed the effect of ultrasound training on gross anatomy knowledge, but previous studies have shown mixed outcomes [4–9].

Some studies have investigated ultrasound and sonoanatomy education for medical students [4–9]. However, only two of these studies have suggested that ultrasound education not only improves medical students’ knowledge of ultrasound and skills but also enhances their anatomical knowledge [4, 8]. The similarities between these two studies are hands-on practice and integration with anatomy courses [4, 8]. The remaining studies showed no statistically significant improvement [5–7]. Interestingly, the studies with hand-on practice showed significant impact [4, 8], but those without ultrasound practice did not have significant results [5–7]. Only one study with hands-on practice showed no significant result, but the ultrasound was taught by undergraduate students [9]. The importance of hands-on practice in ultrasound imaging has been emphasized by other authors as well [11]. These results suggest that learning by doing could be an effective approach to learn anatomy [12], and might be due to concrete experience during practice [13].

However, written assessments in the studies included both ultrasound and gross anatomy images; thus the studies did not analyze the effect of ultrasound on gross anatomy learning individually [4–7]. It is difficult to identify whether the educational gains originated from the improvement of knowledge in ultrasound, gross anatomy, or both. Based on the “learning by doing” theory [12] and the experiential learning theory [13], a Parallel Ultrasound Hands-on (PUSH) course was designed for sonoanatomy. The impact of this course on anatomy learning in both written tests and practical assessments on cadavers was investigated. The hypotheses of our study are the following:

- Hypothesis 1: PUSH training enhances theoretical knowledge of anatomy, evidenced by improved performance in written assessments.
- Hypothesis 2: PUSH training enhances applied knowledge of anatomy, evidenced by improved performance in cadaver laboratory tests.

## Method

### Participants

This crossover study enrolled undergraduate third-year medical students who started to take the anatomy curriculum (the doctor of medicine curriculum in Taiwan includes 4 years of preclinical education and 2 years of clinical training in the hospital). Participating students came from a single institution (Taipei Medical University), located in Taiwan. Participants who had any experiences of ultrasound lectures or hands-on workshops were excluded. This study analyzed the impact of incorporating theoretical and practical ultrasonography training in the preclinical human anatomy curriculum.

### Curriculum design

The Parallel Ultrasound Hands-on (PUSH) course was designed to be complementary to the regular anatomy training that was held throughout a 6-month period. The curricula of these courses (PUSH and traditional anatomy) were developed in parallel, but without direct integration between them (Supplementary Material 1). The PUSH course included seven 40-min lectures (see figure for the course design) held approximately in advance of the formal anatomy class, as well as two hands-on practical workshops. The lecture contents focused on selected structures in the chest and abdomen, which included the heart, hepatobiliary system, urinary system, and great vessels. Only basic ultrasound introduction, ultrasound image and practice techniques were taught in the lecture. In addition, the course included two 120-min workshops (the tutor/learner ratio was 1:4, and all tutors (residents) received the ultrasound faculty training for 6 months in the clinical skill center of WanFang hospital and got the certification) which focused on checklist-guided hands-on practice (Supplementary Material 2), which covered anatomical structures included in the lectures. Each workshop(120 min) focused on two different systems(60 min each). During the workshops, tutors demonstrated first and then students performed hands-on ultrasonographic identification of target anatomical structures on each other. Learners were given enough time to practice ultrasound skills and knowledge acquired during the lectures. Target anatomical structures include cardiovascular, hepatobiliary and urinary systems. Every student had 15mins for hands-on practice and 45mins for observation and discussion with the tutor and scanner in each system. Clear learning goals and tasks were made explicit through an ultrasound checklist provided at the beginning of the course. Students also received real-time feedback from the tutors to ensure that they appropriately identified checklist items.

### Study design

**Fig. 1:**
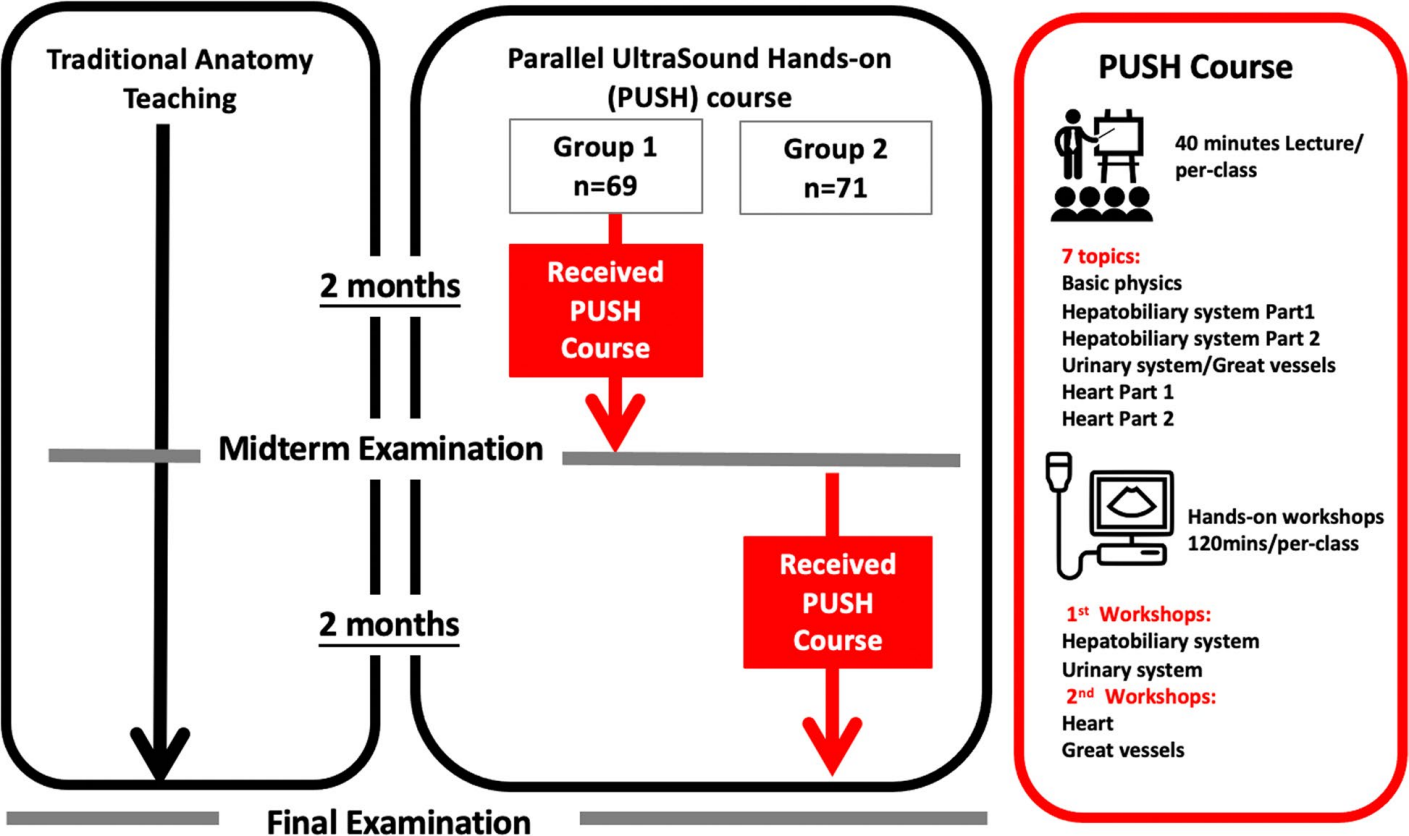
Study design of the PUSH course

Of 164 eligible medical students, 140 (85%) voluntarily participated in this elective course, with no dropout. The percentage of participation was about 91.2%. Students were assigned into two groups based on their schedule availability. One group participated in the PUSH course during the first half of the semester, and the other group participated in the latter half of the semester (Fig. 1). The midterm and final examination scores of the traditional anatomy course were used to measure the educational impact of the PUSH course. The mean scores differences between the two groups in the midterm and final examinations were analyzed. Although the examinations’ blueprints covered the entire human anatomy curriculum, the analysis included only assessment items of anatomical structures included in the PUSH course. The anatomy examination format included written items and laboratory test items on cadavers. The analysis included 50 single-answer multiple choice items from the written test and 50 items on the laboratory test; these items were not specifically associated with ultrasonographic knowledge, performance skills, or images.

### Outcome measurements

In addition to the midterm and final examination scores, the PUSH course included the completion of a learning self-efficacy scale which is designed to measure learners’ confidence in their capability to learn specific subjects (Supplementary Material 3). The item order and descriptions were similar to those of the original published version but were translated to Traditional Mandarin Chinese [14]. The scale consisted of 12 items and covered the cognitive, affective, and psychomotor domains of sonoanatomy training. There were four items in each domain rated with a five-point Likert scale (from 1 to 5; strongly disagree to strongly agree and the neutral value is 3).

### Statistics

An independent t-test was conducted to test for differences in baseline characteristics, written test scores for target anatomy, laboratory examination scores for target anatomy, and learning self-efficacy between the two groups. A dependent sample t-test was employed to test improvement in target anatomy in the written and laboratory tests and stratified the sample by the group. Because t-tests were used in the present study, t-values were also presented based on the t distribution of the obtaining values between groups or paired data. As an absolute t value of a test achieves 1.96, the finding would be statistically significant. Post hoc effect size was calculated based on the formula as follow:

$$Cohen's\;d\;=\;absolute\;\left(\chi_1-\chi_2\right)/squared\;\left(\left(\int1^2-\int2^2\right)/2\right)$$

Here *χ*_1_ refers to mean of correct items in the group 1 and *χ*_2_ refers mean of correct items in the group 2. Besides, $\int1$ indicates standard deviation of correct items in the group 1, and $\int2$ indicates standard deviation of correct items in the group 2.

Because learning self-efficacy was measured in a one-shot survey after the PUSH course, the difference in learning self-efficacy between the two groups was not part of the aims of this study. The scale was used to explore students’ efficacy in learning, the data was analyzed using a critical value test with a neutral score value of 3. If the test value was significantly higher than 3, medical students had positive efficacy in learning sonoanatomy. The analysis included the differences among the three domains of learning self-efficacy after the PUSH course. A general linear model was utilized to compare differences among the domains. If a *P*-value lower than 0.05 was achieved, the outcome reached statistical significance.

## Results

The two groups had similar characteristics, including male and female percentage (chi-square = 0.36; *P* = 0.866) and scores on other anatomy items on the written examination (MD = 1.0; 3.5%; *P* = 0.233) and laboratory examination (MD = 0.6; 2.4%; *P* = 0.515). The only statistically significant difference in test scores between the groups was observed in the midterm written examination. In the first study period, group 1 had a higher written test score for target anatomy (mean correct items = 12.7, 63.5%) than group 2 (mean correct items = 11.2, 55.8%) after the ultrasound course (MD = 1.5(7.6%); *P* = 0.014), but there was no difference in the laboratory examination score for target anatomy between the two groups (mean correct items in group 1 = 11.6, 44.6%; mean correct items in group 2 = 10.9, 41.8%; MD = 0.7(2.8%); *P* = 0.308) (Table 1). In the second study period, group 2 received an ultrasound course and had no significant difference in target anatomy in either the written examination (mean correct items in group 1 = 12.8, 60.9%; mean correct items in group 2 = 12.5, 62.4%; MD = 0.3(1.6%); *P* = 0.472) or the laboratory examination (mean correct items in group 1 = 10.1, 47.9%; mean correct items in group 2 = 9.8, 46.4%; MD = 0.3(1.5%); *P* = 0.592) compared to group 1 (Table 2).

**Table 1:** Primary outcome between two groups on Midterm exam

Objective outcomes	Group 1	Group 2			
(Midterm exam)	Mean ± SD	Mean ± SD	*t*	*d*	*P*
Other anatomy Written examination (30/50)	23.7 ± 4.4	22.7 ± 5.7	1.20	0.20	0.233
Other anatomy Laboratory examination (24/50)	14.7 ± 4.5	14.1 ± 5.8	0.65	0.11	0.515
Target anatomy Written examination^a^ (20/50)	12.7 ± 3.1	11.2 ± 4.0	2.50*	0.43	0.014
Target anatomy Laboratory examination^b^ (26/50)	11.6 ± 3.9	10.9 ± 4.6	1.02	0.17	0.308

^a^ Discrimination *t* = 18.74, ^b^ Discrimination *t* = 24.71; * *P* < 0.05; *d*, Cohen’s *d*; *t*, *t*-value

**Table 2:** Primary outcome between two groups on final exam

Objective outcomes	Group 1	Group 2			
(Final exam)	Mean ± SD	Mean ± SD	*t*	*d*	*P*
Other anatomy Written examination (30/50)	25.0 ± 3.2	24.7 ± 5.2	0.49	0.08	0.627
Other anatomy Laboratory examination (24/50)	19.4 ± 3.9	19.0 ± 5.3	0.55	0.09	0.580
Target anatomy Written examination (20/50)	12.8 ± 2.5	12.5 ± 3.0	0.72	0.12	0.472
Target anatomy Laboratory examination (21/50)	10.1 ± 3.4	9.8 ± 3.5	0.54	0.09	0.592

Furthermore, there was no significant difference in learning self-efficacy of sonoanatomy between the two groups after the PUSH course (Table 3). Overall Cronbach’s alpha was: 0.899 (sub-scales of cognitive domain:0.890, affective domain:0.803, and psychomotor domain: 0.839). Participants provided positive feedback in overall learning self-efficacy after the PUSH course (Mean = 3.68, SD = ±0.56; *P* < 0.001). Similar results were observed in all three subdomains of learning self-efficacy. The students reported positive learning self-efficacy in the cognitive domain (Mean = 3.91, SD = ±0.67; *P* < 0.001), affective domain (Mean = 3.33, SD = ±0.66; *P* < 0.001), and psychomotor domain (Mean = 3.79, SD = ±0.69; *P* < 0.001). Differences in learning self-efficacy among the subdomains were observed. Learning self-efficacy in the cognitive domain was significantly higher than that in the affective domain (MD = 0.58; *P* < 0.001) and psychomotor domain (MD = 0.12; *P* = 0.011). Learning self-efficacy in the affective domain was significantly lower than that in the psychomotor domain (MD = − 0.46; *P* < 0.001) (Table 4).

**Table 3:** Objective outcome between two groups on learning self-efficacy

Subjective outcome	Group 1	Group 2			
(Learning self-efficacy)	Mean ± SD	Mean ± SD	*t*	*d*	*P*
Overall Learning self-efficacy	43.11 ± 7.49	45.08 ± 5.82	−1.66	−0.30	0.100
Learning self-efficacy (Cog.)	3.81 ± 0.78	4.01 ± 0.53	−1.68	−0.30	0.095
Learning self-efficacy (Aff.)	3.29 ± 0.68	3.37 ± 0.65	−0.66	−0.12	0.510
Learning self-efficacy (Psy.)	3.68 ± 0.77	3.89 ± 0.60	−1.77	−0.32	0.079

* *P* < 0.05, *Aff.* affective domain, *Cog.* cognitive domain, *d* Cohen’s *d*, *LSE* learning self-efficacy, *M* mean, *Psy.* psychomotor domain, *SD* standard deviation, *t t*-value

**Table 4:** Summary of analyses of learning self-efficacy at the end of semester

			Multivariate test ^b^			
	Critical value test ^a^		Compared to Aff.		Compared to Psy.	
L-SES	*Mean ± SD*	*d*	*MD*	*P*	*MD*	*P*
Overall	3.68 ± 0.56**	2.41	–	–	–	–
Cog.	3.91 ± 0.67**	2.78	0.58	< 0.001	0.12	0.011
Aff.	3.33 ± 0.66**	1.00	–	–	−0.46	< 0.001
Psy.	3.79 ± 0.69**	2.28	–	–	–	–

^a^, one-sample *t*-test with threshold value 3; ^b^, repeated measurement; ** *P* < 0.001; *Aff.* affective domain, *Cog.* cognitive domain, *d* Cohen’s *d*, *LSE* learning self-efficacy, *MD* mean difference, *Psy.* psychomotor domain, *SD* standard deviation

## Discussion

### The gap between ultrasound images and the gross anatomy

The results of this study determined that the group receiving PUSH course intervention increased scores on the written test in the midterm examination but led to no significant improvement in the laboratory examination in gross anatomy. This result may be due to the vast difference between ultrasound images and the actual appearance of gross anatomy in the cadaver. Generally, ultrasound helps students learn the location and relationships between anatomical structures and their disposition in a living human body, strengthening their cognition and concepts of anatomy [15]. Additionally, medical students can observe dynamic changes in the heart and the blood flow of vessels through ultrasound. Ultrasonography allows medical students to learn anatomy from a different point-of-view. However, these advantages might not be applicable to the recognition of organs or structures of cadaveric origin. There is a large gap between monochromatic images and the appearance of cadaveric organs or structures. The results from the previous studies suggest that ultrasonographic training can aid learners’ understanding of anatomical concepts (such as the three-dimensional orientation and spatial correlation of anatomical structures within the body), which may complement and enhance the traditional anatomical curriculum [16, 17].

Several institutions integrate ultrasound training in general medical education, including anatomy and physical examination [18]. Many studies have revealed positive findings for student satisfaction, but the learning outcomes of anatomy via ultrasound curricula are controversial [4, 5, 9, 19, 20]. Many of these studies lacked control or pre-intervention groups in learning outcome evaluation. There is still insufficient evidence to suggest that ultrasound training leads to significant improvement in anatomical knowledge or physical examination skills of undergraduate medical students [18]. This study attempted to overcome these gaps by utilizing a crossover study design and created an additional ultrasound course that was independent of the current anatomy curriculum. We found that by participating in an elective ultrasound course, undergraduate medical students were able to improve their midterm but not final anatomy written examination scores.

### The reasons for the effect of PUSH on anatomy learning

There may be several reasons for the findings of this study. First, the results of learning self-efficacy at the end of the semester showed the cognitive domain is significantly higher than that in the psychomotor domain. The early exposure of medical students to clinical tools can increase their learning motivation and interest because it enables them to understand how to apply their anatomy knowledge to clinical practice by learning ultrasound [21, 22], and this creates awareness of the importance of anatomy in the preclinical training years. Bridging anatomy knowledge and clinical practice is important and can enable medical students to understand the need for a functional understanding of anatomy [5, 21]. Ultrasonographic training also provides a clinical context to justify the need for anatomical knowledge, making anatomy more concrete and practical [5].

Second, the use of ultrasound to support anatomy instruction allows students to observe the dynamic changes in organs, such as the opening and closing of heart valves, the direction of blood flow and the importance of heart physiology features, such as ejection fraction and cardiac output. This allows learners to gain more insight of the application of anatomy to the understanding of clinical medicine. Third, medical students learn to operate ultrasound probes to identify relevant anatomical structures through hands-on ultrasonographic training. This process enables learners to develop a three-dimensional understanding of the disposition of specific structures in the body and their spatial relationships.

### Ultrasound training improves anatomy learning

Previous studies showed varied outcomes in exploring the efficacy of learning gross anatomy via ultrasound [4–9]. It is difficult to reach specific conclusions because of significant differences in curricular design. By reviewing previously published studies, a new curriculum was designed and implemented, yielding positive educational outcomes. The main features of this curriculum were a hands-on practical approach in a small group setting, and included a crossover study design to compare the educational outcome. Most studies did not include a hands-on component in their ultrasound course design [5–7] and none of them provided students with the opportunity to develop ultrasound skills by concrete experience. Conducting ultrasonographic examinations on real humans strongly strengthens the three-dimensional perception of human anatomy [23]. To minimize any negative effects of elective ultrasonography training on the original compulsory gross human anatomy course, the PUSH course was conducted in a manner that did not affect the time allotted to the study of gross anatomy. Many studies assessed ultrasound ability and knowledge in tests and did not report these results separately [4–7]. Consequently, it is unclear whether their observations were due to improvements in anatomy knowledge or ultrasound knowledge. It may be taken for granted that the scores of ultrasound knowledge would improve after learning ultrasound compared to the scores of learners who did not have this opportunity. Hence, to focus on the effect of learning anatomy itself, this study did not include any specific ultrasound imaging or knowledge evaluation. Only anatomy written tests and cadaver laboratory tests were conducted. Holding a parallel curriculum could improve students’ acquisition of anatomical knowledge with the aid of ultrasound training.

### Characteristics of the PUSH course

These findings may be related to several specific characteristics of the PUSH course. The course methodology had four particular features. First, learners participated voluntarily in the course because they could gain early exposure to clinical medicine that would be valuable for their future and increased their motivation. Second, during the hands-on workshops, learners were given enough time to practice ultrasound skills and knowledge acquired during the lectures. This meant that these students could adopt learning strategies depending on their educational needs and preferences [24]. Third, clear learning goals and tasks were made explicit through an ultrasound checklist provided at the beginning of the course, which allowed learners to prepare before the workshops. Fourth, on-site tutors corrected students’ manipulation of ultrasound probes and ensured that they appropriately identified checklist items through synchronous observation and feedback.

These features are different from those of previous studies on ultrasound integrated with anatomy [3–7, 9]. Therefore, these findings suggest that in the future, integrating ultrasound and anatomy courses requires careful consideration of the course design, including self-directed learning, as well as checklist-oriented and hands-on workshops with on-site tutors [25]. Traditionally, ultrasound learning was provided exclusively during the clinical formative years, but the results of this study suggest that ultrasonographic training is a feasible form of early clinical exposure that can not only motivate learners but help them bridge the gap between preclinical training to the practice of medicine.

### Limitations of the study

This study had some limitations. First, the blueprint of the cadaveric laboratory assessment was not specifically aligned with the ultrasound curriculum, therefore, the cadaveric laboratory test improvement observed in this study may not be directly related to the ultrasonographic training. Second, students received ultrasound checklists at the beginning of the course and tutors were available for immediate feedback during hands-on practice, but we did not evaluate students’ ultrasound learning outcomes systematically. Third, it is hard to ascertain how the additional lectures that students received as part of the PUSH may have influenced the test results as they presumably contained reviews of the anatomy, thus confounding the true impact of the ultrasound intervention. Fourth, 140 of 164 learners who joined the study were all volunteers, which may contribute to self-selection bias. Because students who come to participate voluntarily may be more motivated, and the results of this study cannot be applied to the entire student population. These suggest that future studies should attempt to avoid these limitations.

## Conclusion

Overall, the PUSH course with its active learning played a supportive role in learning anatomy and responded to the trends in clinical practice, in which ultrasound is becoming a common tool of the modern clinician. The findings of this study suggest that the implementation of a sonoanatomy course enhanced their learning in the field of anatomy.

## Supplementary Information

**Additional file 1.**

**Additional file 2.**

**Additional file 3.**

## Data Availability

The datasets used and/or analyzed during the current study are available from the corresponding author on reasonable request.

## Abbreviations

PUSH: Parallel UltraSound Hands-on
MD: Mean Difference
SD: Standard Deviation
