# Supplementary material for: Does ultrasound education improve anatomy learning? Effects of the Parallel Ultrasound Hands-on (PUSH) undergraduate medicine course
Source: BMC Med Educ. 2022 Mar 27;22:207. doi: 10.1186/s12909-022-03255-4 (PMC8962240; doi:10.1186/s12909-022-03255-4)
Supplement: Supplementary file 1 — Additional file 1. [file 12909_2022_3255_MOESM1_ESM.doc]

**Supplementary material 1 : about traditional anatomy education in Taiwan**

In the second semester of the second-year medical school, the students will study the systemic integration course of anatomy and physiology (skeletal muscle system, respiratory and circulatory system, nervous system, digestive system, genitourinary and endocrine system). Lectures are the main method to introduce systemic anatomy. The total number of lecture hours is 104 hours. In the first semester of the third-year medical school, regional anatomy is conducted and includes 32 hours of lectures, and donated cadavers are used for 96 hours of anatomy laboratory. The total curriculum of anatomy experiments is as **Table.**

Textbooks, including Gray’s Anatomy Students and Grant’s dissector, are the main resources for anatomical learning.

The assessment method is based on the written examination at mid and final terms (50 multiple-choice questions) (50%), laboratory examination (50items, 50%).

**Third-year Medical Student Anatomy Experiments Curriculum**

Mon、Wed of Each week 13:10—17:00 Total 13 weeks except holidays and review days

| **Traditional Anatomy Curriculum** | | | | | | **PUSH Course** |
| --- | --- | --- | --- | --- | --- | --- |
| Week | **Head** | **Neck/Thorax/Back** | **Abdomen/Pelvis** | **Upper Extremities** | **Lower Extremities** |  |
| 1 | 1. Surface anatomy  2. Bony structures of head | Skin incision and Superficial region of neck and thorax (I) | Superficial fascia of anterior abdominal wall | Cutaneous structures of upper limb (I) | 1. Surface anatomy & bony structures of anterior lower limb 2. Skin incision of anterior lower limb |  |
| 1 | 1. Skin incision of face  2. Cutaneous ns. of trigeminal nerve | Superficial region of neck and thorax (II) | 1. Skeleton of the pelvis 2. Muscles of anterior abdominal wall | Cutaneous structures of upper limb (II) | Superficial structures of anterior lower limb (I) | Basic Physics |
| 2 | Muscles & blood vessels of face (I) | Pectoral region | 1. Reflection of the abdominal wall 2. Peritoneum and peritoneal cavity | Axilla (I) | Superficial structures of anterior lower limb (II) |  |
| 2 | Muscles & blood vessels of face (II) | Anterolateral thoracic wall | Removal of the gastrointestinal tract | Axilla (II) | Anterior compartment of thigh (I) | Hepatobiliary part 1 |
| 3 | Muscles & blood vessels of face (III) | Thoracic cavity | Posterior abdominal viscera | Axilla (III) | Anterior compartment of thigh (II) | Hepatobiliary part 2 |
| 3 | Parotid region & facial nerve | Removal of heart and lungs | Posterior abdominal wall | Arm (I) | Medial compartment  of thigh | Urinary System ＆  Hands-on Workshop |
| 4 | Temporal Region | Posterior mediastinum and posterior thoracic wall | Diaphragm | Arm (II) | Quiz (I) | Great vessels |
| 4 | 1. Infra-temporal fossa (I)  2. Temporal mandibular joint (TMJ) | Anterior triangle of neck | External genitalia and perineum | Anterior compartment  of forearm (I) | Lateral compartment  of leg | Heart part 1 |
| 5 | Infra-temporal fossa (II) | Posterior triangle of neck | Urogenital triangle | Anterior compartment  of forearm (II) | Anterior compartment  of leg | Heart part 2 &  Hands-on Workshop |
| 5 | Orbit (I) – anterior view & extraocular muscles and eyeball | Root of neck (I) | Pelvic cavity (I) | Anterior compartment  of forearm (III) | Dorsum of foot |  |
| 6 | Orbit (II) – anterior view (blood vessels and nerve) | Root of neck (II) | Internal iliac artery (I) | Anterior compartment  of forearm (IV) | Quiz (II) |  |
| 6 | **Mid-Term Test** | **Written Test+ Laboratory Test** | | | |  |
| 7 | The scalp & removal of the calvaria | Skin incision and  Superficial back | 1. Anal triangle 2. Celiac trunk, stomach, spleen, liver, and gall bladder | Superficial back (I) | 1. Surface anatomy & bony structures of posterior lower limb  2. Skin incision of posterior lower limb (I) |  |
| 7 | Cranial meninges & dura sinuses | Deep back and Suboccipital region I | Superior mesenteric artery and small intestine | Superficial back (II) | 1. Skin incision of posterior lower limb (I) 2. Superficial structures of posterior lower limb | Basic Physics |
| 8 | Removal of the brain | Deep back and Suboccipital region II | Inferior mesenteric artery and large intestine | Shoulder region (I) | Gluteal region (I) |  |
| 8 | Cranial fossa & dissection of brain | Vertebrae and spinal cord | Duodenum, pancreas, and hepatic portal vein | Shoulder region (II) | Gluteal region (II) | Hepatobiliary part 1 |
| 9 | Orbits (III) – superior view | Heart and lungs | Bisection of pelvic cavity | Forearm extensor (I) | 1. Posterior compartment of thigh  2. Popliteal fossa | Hepatobiliary part 2 |
| 9 | Orbits (IV) – superior view | Superior mediastinum | Pelvic cavity (II)  Internal iliac artery(II) | Forearm extensor (II) | Posterior compartment of leg (I) | Urinary System &  Hands-on Workshop |
| 10 | Bisection and removal of head | Deep neck | Urinary bladder, rectum, and anal canal | Palm (I) | Quiz (III) | Great vessels |
| 10 | Nasal cavity & pharynx | Cervical viscera (I) | Sacral plexus (I) | Palm (II) | Posterior compartment of leg (II) | Heart part 1 |
| 11 | Pterygopalatine fossa | Cervical viscera (II) | Sacral plexus (II) | Palm (III) | Sole of foot (I) | Heart part 2 &  Hands-on Workshop |
| 11 | Oral cavity: mouth & palate | Submandibular region (I) | Pelvic diaphragm (I) | Palm (IV) | Sole of foot (II) |  |
| 12 | Oral cavity: tongue & salivary glands | Submandibular region (II) | Pelvic diaphragm (II) | Joints of the upper  limb (I) | Joints of lower limb |  |
| 12 | Middle ear | **Review** | **Review** | Joints of the upper  limb (II) | Quiz (IV) |  |
| 13 | **Final-Term Test** | **Written Test+ Laboratory Test** | | | |  |

PUSH course: Parallel UltraSound Hands-on course

Reference：1. Gray’s Anatomy for Student. 2. Grant’s Dissector.
