# Supplementary material for: Does ultrasound education improve anatomy learning? Effects of the Parallel Ultrasound Hands-on (PUSH) undergraduate medicine course
Source: BMC Med Educ. 2022 Mar 27;22:207. doi: 10.1186/s12909-022-03255-4 (PMC8962240; doi:10.1186/s12909-022-03255-4)
Supplement: Supplementary file 2 — Additional file 2. [file 12909_2022_3255_MOESM2_ESM.docx]

**Supplementary material 2: Sonoanatomy Checklist**

Sonoanatomy Checklist

Introduction + FAST (Focus Assessment of Sonography for Trauma)

| Introduction | Check |
| --- | --- |
| Patient preparation |  |
| Doctor preparation |  |
| Check probe/monitor marker/orientation |  |
| Adjust Gain/Depth/  Body maker/Freeze/Printer/measurement/TGC |  |
| B-mode/M-mode/D-mode/B/B mode |  |
| Manipulation: Sliding/Rocking/Tilting/Rotating/Compression |  |
| FAST | Check |
| RUQ: pleural base above and below diaphragm |  |
| RUQ: hepato-renal fossa (Morison’s pouch) |  |
| LUQ: pleural base above and below diaphragm |  |
| LUQ: splenorenal recess |  |
| Pelvis: CDS or recto-vesicular pouch (Transverse) |  |
| Pelvis: CDS or recto-vesicular pouch (Sagittal) |  |
| Subcostal: pericardial cavity (identify RV/LV) |  |
| Parasternal long: pericardial cavity (identify RV/LV) |  |

Great Vessels

| Vessels | Check |
| --- | --- |
| Abdominal aorta: transverse view to bifurcation |  |
| Abdominal aorta: Sagittal view (celiac trunk/SMA) |  |
| Inferior vena cava: long axis/short/diameter(RA) |  |
| Deep Vein Scan: femoral A/V(L/S), popliteal A/V |  |
| Deep Vein Scan: compression test |  |

Urinary System

| Urology | Check |
| --- | --- |
| Right kidney: cortex/medulla/renal pelvis (L/S) |  |
| Left kidney: cortex/medulla/renal pelvis (L/S) |  |
| Bladder: Long/Short axis |  |
| Prostate: size/seminal vesicles |  |

Hepatobiliary system

| Hepatobiliary system | Check |
| --- | --- |
| Hepatorenal fossa (Morison’s pouch) |  |
| Gallbladder: long axis/short axis |  |
| Gallbladder: check wall thickness/content |  |
| Portal vein: Left/Right/Main |  |
| Hepatic vein: R/M/L/to IVC |  |
| Portal triad: portal vein/hepatic artery/biliary tract |  |
| Liver Segment: S1-S8 (optional) |  |
| Spleen: Long axis/Short axis |  |

Heart

| Heart-Subcostal view | Check |
| --- | --- |
| Pericardial cavity |  |
| Check RV/LV |  |
| Inferior vena cava(location/diameter measurement) |  |
| Heart-Parasternal Long Axis | Check |
| Pericardial cavity |  |
| Right Ventricle: size/wall |  |
| Left Ventricle: size/wall/contractility |  |
| Left Ventricle: Ejection fraction |  |
| Aorta root: Location/Diameter |  |
| Valve movement: Mitral/tricuspid/Aorta(optional) |  |
| Thoracic Aorta(optional) |  |
| Heart-Parasternal Short Axis | Check |
| Papillary Muscle Level: papillary muscle(RV/LV) |  |
| Mitral Valve Level: Mitral valve/contractility |  |
| Aortic Valve Level: RA/RV/PA/LA/Aorta |  |
| Heart-Apical Four/Five View | Check |
| LV/RV/LA/RA |  |
| Mitral valve/Tricuspid valve |  |
| Aorta root/Aorta valve |  |
| Ventricle wall identification(optional) |  |
