# Supplementary material for: Does ultrasound education improve anatomy learning? Effects of the Parallel Ultrasound Hands-on (PUSH) undergraduate medicine course
Source: BMC Med Educ. 2022 Mar 27;22:207. doi: 10.1186/s12909-022-03255-4 (PMC8962240; doi:10.1186/s12909-022-03255-4)
Supplement: Supplementary file 3 — Additional file 3. [file 12909_2022_3255_MOESM3_ESM.docx]

**Supplementary material 3: Learning Self-Efficacy Scale**

| **Domain/No.** | **Disagree** *<***- - - -***>* **agree** | | | | | |
| --- | --- | --- | --- | --- | --- | --- |
|  | **Item** | 1 | 2 | 3 | 4 | 5 |
| **Cognitive** |  |  |  |  |  |  |
| 1 | I can recall how to perform “ultrasound”. | 1 | 2 | 3 | 4 | 5 |
| 2 | I understand the content of “ultrasound” and can demonstrate it to others. | 1 | 2 | 3 | 4 | 5 |
| 3 | I can verbally explain the purpose and principle of operating “ultrasound”. | 1 | 2 | 3 | 4 | 5 |
| 4 | I can verbally explain the sequence and interrelationship between each step. | 1 | 2 | 3 | 4 | 5 |
| **Affective** |  |  |  |  |  |  |
| 5 | I think I spend more time on “this” course than on others. | 1 | 2 | 3 | 4 | 5 |
| 6 | I think I gain more in “this” course than in others. | 1 | 2 | 3 | 4 | 5 |
| 7 | I tend to pay more attention to information related to “this” course. | 1 | 2 | 3 | 4 | 5 |
| 8 | I tend to actively look for information related to “this” course. | 1 | 2 | 3 | 4 | 5 |
| **Psychomotor** |  |  |  |  |  |  |
| 9 | I can precisely imitate the instructor’s steps and actions of “ultrasound”. | 1 | 2 | 3 | 4 | 5 |
| 10 | I can smoothly complete the operation steps of “ultrasound”. | 1 | 2 | 3 | 4 | 5 |
| 11 | I try to monitor my “ultrasound” for improvements. | 1 | 2 | 3 | 4 | 5 |
| 12 | I try to monitor my “ultrasound” operations and make proper adjustments as needed. | 1 | 2 | 3 | 4 | 5 |

Reference: Kang YN, Chang CH, Kao CC, Chen CY, Wu CC. Development of a short and universal learning self-efficacy scale for clinical skills. *PLoS One*. 2019;14(1):e0209155. Published 2019 Jan 7. doi:10.1371/journal.pone.0209155
